# A monoclinic polymorph of 1-(4-chloro­phen­yl)-3-(4-methoxy­phen­yl)prop-2-en-1-one

**DOI:** 10.1107/S1600536809054956

**Published:** 2010-01-09

**Authors:** Jerry P. Jasinski, Ray J. Butcher, B. Narayana, S. Samshuddin, H. S. Yathirajan

**Affiliations:** a Department of Chemistry, Keene State College, 229 Main Street, Keene, NH 03435-2001, USA; b Department of Chemistry, Howard University, 525 College Street NW, Washington, DC 20059, USA; c Department of Studies in Chemistry, Mangalore University, Manalaganotri 574 199, India; d Department of Studies in Chemistry, University of Mysore, Manasagangotri, Mysore 570 006, India

## Abstract

The crystal structure of the title compound, C_16_H_13_ClO_2_ (II), (space group *P*2_1_/*c*,) is a polymorph of the structure, (I), reported by Harrison, Yathirajan, Sarojini, Narayana & Indira [*Acta Cryst.* (2006), E**62**, o1647–o1649] in the ortho­rhom­bic space group *Pna*2_1_. The dihedral angle between the mean planes of the 4-chloro- and 4-meth­oxy-substituted benzene rings is 52.9 (1)° in (II) compared to 21.82 (6)° for polymorph (I). The dihedral angles between the mean planes of the prop-2-en-1-one group and those of the 4-chloro­phenyl and 4-methoxy­phenyl rings are 23.3 (3) and 33.7 (1)°, respectively. in (II). The corresponding values are 17.7 (1) and 6.0 (3)°, respectively, in polymorph (I). In the crystal, weak C—H⋯π inter­actions are observed.

## Related literature

For the orthorhomic polymorph, see: Harrison *et al.* (2006 ▶). For the biological activity of chalcones and flavonoids, see: Dimmock *et al.* (1999 ▶); Opletalova & Sedivy (1999 ▶); Lin *et al.* (2002 ▶); Nowakowska (2007 ▶). For the synthesis and biological activity of some fluorinated chalcone derivatives, see: Nakamura *et al.* (2002 ▶). For non-linear optical studies of chalcones and their derivatives, see: Sarojini *et al.* (2006 ▶); Poornesh *et al.* (2009 ▶); Shettigar *et al.* (2006 ▶, 2008 ▶). For our studies of chalcones, see: Jasinski *et al.* (2009 ▶).
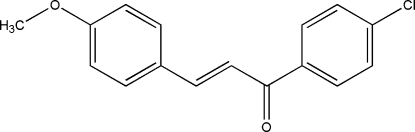


## Experimental

### 

#### Crystal data

C_16_H_13_ClO_2_
*M* *_r_* = 272.71
Monoclinic, 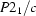

*a* = 15.6695 (7) Å
*b* = 14.1235 (8) Å
*c* = 5.8455 (3) Å
β = 90.771 (5)°
*V* = 1293.53 (12) Å^3^
*Z* = 4
Cu *K*α radiation
μ = 2.57 mm^−1^
*T* = 110 K
0.54 × 0.13 × 0.08 mm

#### Data collection

Oxford Diffraction Xcalibur diffractometer with a Ruby (Gemini Cu) detector
Absorption correction: multi-scan (*CrysAlis RED*; Oxford Diffraction, 2007 ▶) *T*_min_ = 0.483, *T*_max_ = 0.558
5083 measured reflections
2537 independent reflections
2223 reflections with *I* > 2σ(*I*)
*R*_int_ = 0.021

#### Refinement

*R*[*F* ^2^ > 2σ(*F* ^2^)] = 0.035
*wR*(*F* ^2^) = 0.096
*S* = 1.04
2537 reflections
173 parameters
H-atom parameters constrained
Δρ_max_ = 0.25 e Å^−3^
Δρ_min_ = −0.22 e Å^−3^

### 

Data collection: *CrysAlis PRO* (Oxford Diffraction, 2007 ▶); cell refinement: *CrysAlis RED* (Oxford Diffraction, 2007 ▶); data reduction: *CrysAlis RED*; program(s) used to solve structure: *SHELXS97* (Sheldrick, 2008 ▶); program(s) used to refine structure: *SHELXL97* (Sheldrick, 2008 ▶); molecular graphics: *SHELXTL* (Sheldrick, 2008 ▶); software used to prepare material for publication: *SHELXL97*.

## Supplementary Material

Crystal structure: contains datablocks global, I. DOI: 10.1107/S1600536809054956/om2306sup1.cif

Structure factors: contains datablocks I. DOI: 10.1107/S1600536809054956/om2306Isup2.hkl

Additional supplementary materials:  crystallographic information; 3D view; checkCIF report

## Figures and Tables

**Table 1:** Hydrogen-bond geometry (Å, °) *Cg*1 and *Cg*2 are the centroids of the C1–C6 and C10–C15 rings, respectively.

*D*—H⋯*A*	*D*—H	H⋯*A*	*D*⋯*A*	*D*—H⋯*A*
C2—H2⋯*Cg*1^i^	0.95	2.85	3.4675 (15)	124
C12—H12⋯*Cg*2^ii^	0.95	2.92	3.6616 (17)	136

Symmetry codes: (i) 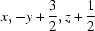; (ii) 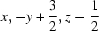.

**Fig. 1.**
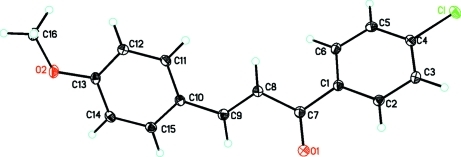
Molecular structure of the title compound, C16H13ClO2, (II), showing the atom labeling scheme and 50% probability displacement ellipsoids.

**Fig. 2.**
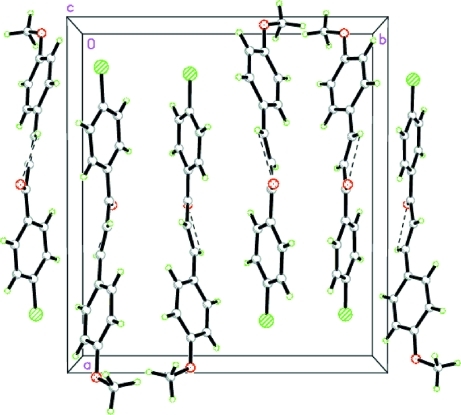
Packing diagram of the title compound, (II), viewed down the c axis. Weak C–H..O intramolecular hydrogen bond interactions are shown as dashed lines.

## supplementary crystallographic information

### Comment

Chalcone is an unique template molecule that is associated with several biological activities. A review on the bioactivities of chalcones is described (Dimmock *et al.* 1999). Chalcones and their heterocyclic analogs as potential antifungal chemotherapeutic agents is published (Opletalova & Sedivy, 1999). Chalcones and flavonoids as anti-tuberculosis agents has been reported (Lin *et al.* 2002) and a review of anti-infective and anti-inflammatory chalcones is also described (Nowakowska, 2007) as well as the synthesis and biological activities of some fluorinated chalcone derivatives (Nakamura *et al.* 2002). In addition, chalcones are finding applications as organic non-linear optical materials (NLO) due to their good SHG conversion efficiencies (Sarojini *et al.* 2006). Recently, non-linear optical studies on a few chalcones and their derivatives were reported (Poornesh *et al.* 2009; Shettigar *et al.* 2006; 2008). In continuation of our work on chalcones (Jasinski *et al.* 2009) and in view of the importance of chloro chalcones, this paper describes a new polymorphic form of (I), C_16_H_13_ClO_2_, 1-(4-chlorophenyl)-3-(4-methoxyphenyl)-prop-2-en-1-one, first reported by Harrison *et al.* (2006). Substantial changes in the cell parameters provides solid support for the recognition of this new polymorphic form for (I).

The title compound, (II), is a chalcone derivative with 4-chlorophenyl and 4-methoxyphenyl rings bonded at the opposite ends of a propenone group, the biologically active region (Fig.1). The dihedral angle between mean planes of the 4-chloro and 4-methoxy substituted benzene rings in (II) is 52.9 (1)° compared to 21.82 (6)° (Harrison *et al.* (2006); 4-chlorohenyl & 4-methoxyphenyl) for polymorph (I) in the orthorhombic, *Pna2_1_*, space group. The angles between the mean plane of the prop-2-ene-1-one group and those of the 4-chlorophenyl and 4-methoxyphenyl rings in (II) are 23.3 (3)° and 33.7 (1)°, respectively. This compares to 17.7 (1)° and 6.0 (3)° in polymorph (I). A weak intramolecular C9–H9···O1 hydrogen bond interaction is present which may help to maintain the molecular conformation of the molecule (Table 1) and similar to that observed in (I). While no classical hydrogen bonds are present, weak intermolecular C–H···*Cg*π-ring interactions are observed, Cg1 = C1–C6 and Cg2 = C10–C15, see Table 1.

### Experimental

In (II), 4-chloroacetophenone in ethanol (1.54 g, 0.01 mol) (25 ml) was mixed with 4-methoxybenzaldehyde (1.36 g, 0.01 mol) in ethanol (25 ml) and the mixture was treated with an aqueous solution of potassium hydroxide (20 ml, 5%). This mixture was stirred well and left to stand for 24 hr. The resulting crude solid mass was collected by filtration and recrystallized from ethanol, yielding clear blocks of (II). Yield: 90%, m.p.: 391–393 K, analysis found (calculated) for C_16_H_13_ClO_2_: C: 70.5 (70.4%); H: 4.72 (4.76%). The preparation and crystallization procedure for (I) was identical to that described above for (II). However, in (I) the m.p. measured 380 K, a difference of 12 K. The samples of (I) and (II) were not independently tested for concomitant polymorphism.

### Refinement

All of the H atoms were placed in their calculated positions and then refined using the riding model with C—H = 0.95–0.98 Å, and with *U*_iso_(H) = 1.18–1.48*U*_eq_(C).

### Crystal data

**Table:**

C_16_H_13_ClO_2_	*F*(000) = 568
*M**_r_* = 272.71	*D*_x_ = 1.400 Mg m^−^^3^
Monoclinic, *P*2_1_/*c*	Cu *K*α radiation, λ = 1.54184 Å
Hall symbol: -P 2ybc	Cell parameters from 3121 reflections
*a* = 15.6695 (7) Å	θ = 4.2–73.8°
*b* = 14.1235 (8) Å	µ = 2.57 mm^−^^1^
*c* = 5.8455 (3) Å	*T* = 110 K
β = 90.771 (5)°	Needle, colorless
*V* = 1293.53 (12) Å^3^	0.54 × 0.13 × 0.08 mm
*Z* = 4	

### Data collection

**Table:**

Oxford Diffraction Xcalibur diffractometer with a Ruby (Gemini Cu) detector	2537 independent reflections
Radiation source: Enhance (Cu) X-ray Source	2223 reflections with *I* > 2σ(*I*)
graphite	*R*_int_ = 0.021
Detector resolution: 10.5081 pixels mm^-1^	θ_max_ = 74.0°, θ_min_ = 4.2°
ω scans	*h* = −19→17
Absorption correction: multi-scan (*CrysAlis RED*; Oxford Diffraction, 2007)	*k* = −16→17
*T*_min_ = 0.483, *T*_max_ = 0.558	*l* = −5→7
5083 measured reflections	

### Refinement

**Table:**

Refinement on *F*^2^	Primary atom site location: structure-invariant direct methods
Least-squares matrix: full	Secondary atom site location: difference Fourier map
*R*[*F*^2^ > 2σ(*F*^2^)] = 0.035	Hydrogen site location: inferred from neighbouring sites
*wR*(*F*^2^) = 0.096	H-atom parameters constrained
*S* = 1.04	*w* = 1/[σ^2^(*F*_o_^2^) + (0.0574*P*)^2^ + 0.4041*P*] where *P* = (*F*_o_^2^ + 2*F*_c_^2^)/3
2537 reflections	(Δ/σ)_max_ = 0.001
173 parameters	Δρ_max_ = 0.25 e Å^−^^3^
0 restraints	Δρ_min_ = −0.22 e Å^−^^3^

### Special details

**Table:**

Geometry. All e.s.d.'s (except the e.s.d. in the dihedral angle between two l.s. planes) are estimated using the full covariance matrix. The cell e.s.d.'s are taken into account individually in the estimation of e.s.d.'s in distances, angles and torsion angles; correlations between e.s.d.'s in cell parameters are only used when they are defined by crystal symmetry. An approximate (isotropic) treatment of cell e.s.d.'s is used for estimating e.s.d.'s involving l.s. planes.
Refinement. Refinement of *F*^2^ against ALL reflections. The weighted *R*-factor *wR* and goodness of fit *S* are based on *F*^2^, conventional *R*-factors *R* are based on *F*, with *F* set to zero for negative *F*^2^. The threshold expression of *F*^2^ > σ(*F*^2^) is used only for calculating *R*-factors(gt) *etc*. and is not relevant to the choice of reflections for refinement. *R*-factors based on *F*^2^ are statistically about twice as large as those based on *F*, and *R*- factors based on ALL data will be even larger.

### Fractional atomic coordinates and isotropic or equivalent isotropic displacement parameters (Å^2^)

**Table:**

	*x*	*y*	*z*	*U*_iso_*/*U*_eq_	
Cl1	0.84683 (2)	0.61936 (3)	0.17788 (6)	0.02523 (13)	
O1	0.47093 (7)	0.63259 (9)	0.70069 (19)	0.0289 (3)	
O2	0.01821 (7)	0.61488 (8)	−0.07719 (19)	0.0241 (3)	
C1	0.57310 (9)	0.62821 (10)	0.4074 (3)	0.0181 (3)	
C2	0.63878 (9)	0.66090 (10)	0.5520 (2)	0.0188 (3)	
H2	0.6255	0.6849	0.6990	0.023*	
C3	0.72286 (9)	0.65859 (10)	0.4828 (3)	0.0198 (3)	
H3	0.7672	0.6817	0.5800	0.024*	
C4	0.74128 (9)	0.62174 (10)	0.2682 (3)	0.0193 (3)	
C5	0.67775 (10)	0.58747 (11)	0.1233 (3)	0.0205 (3)	
H5	0.6916	0.5612	−0.0212	0.025*	
C6	0.59317 (9)	0.59211 (11)	0.1929 (3)	0.0202 (3)	
H6	0.5489	0.5705	0.0934	0.024*	
C7	0.48352 (9)	0.63066 (10)	0.4945 (3)	0.0200 (3)	
C8	0.41225 (9)	0.63059 (11)	0.3255 (3)	0.0211 (3)	
H8	0.4223	0.6467	0.1704	0.025*	
C9	0.33359 (9)	0.60773 (11)	0.3930 (3)	0.0200 (3)	
H9	0.3284	0.5885	0.5479	0.024*	
C10	0.25468 (9)	0.60893 (10)	0.2562 (3)	0.0181 (3)	
C11	0.24715 (9)	0.65496 (11)	0.0440 (3)	0.0202 (3)	
H11	0.2960	0.6839	−0.0206	0.024*	
C12	0.16938 (9)	0.65893 (11)	−0.0730 (3)	0.0200 (3)	
H12	0.1648	0.6915	−0.2149	0.024*	
C13	0.09809 (9)	0.61476 (10)	0.0193 (3)	0.0187 (3)	
C14	0.10492 (9)	0.56679 (11)	0.2274 (3)	0.0202 (3)	
H14	0.0567	0.5354	0.2882	0.024*	
C15	0.18181 (9)	0.56513 (11)	0.3443 (2)	0.0199 (3)	
H15	0.1856	0.5336	0.4877	0.024*	
C16	0.00342 (10)	0.67344 (13)	−0.2722 (3)	0.0263 (3)	
H16A	0.0140	0.7397	−0.2311	0.039*	
H16B	−0.0559	0.6662	−0.3248	0.039*	
H16C	0.0420	0.6547	−0.3948	0.039*	

### Atomic displacement parameters (Å^2^)

**Table:**

	*U*^11^	*U*^22^	*U*^33^	*U*^12^	*U*^13^	*U*^23^
Cl1	0.0180 (2)	0.0276 (2)	0.0302 (2)	0.00214 (13)	0.00556 (14)	0.00128 (15)
O1	0.0214 (6)	0.0441 (7)	0.0212 (6)	−0.0035 (5)	0.0006 (4)	0.0008 (5)
O2	0.0158 (5)	0.0266 (6)	0.0298 (6)	−0.0025 (4)	−0.0041 (4)	0.0048 (5)
C1	0.0174 (7)	0.0170 (7)	0.0198 (7)	−0.0009 (5)	−0.0019 (5)	0.0039 (6)
C2	0.0197 (7)	0.0188 (7)	0.0180 (7)	0.0019 (5)	−0.0009 (5)	−0.0002 (6)
C3	0.0185 (7)	0.0188 (7)	0.0221 (7)	0.0000 (5)	−0.0031 (5)	−0.0009 (6)
C4	0.0167 (7)	0.0172 (7)	0.0239 (7)	0.0018 (5)	0.0022 (6)	0.0034 (6)
C5	0.0251 (7)	0.0189 (7)	0.0176 (7)	0.0019 (6)	0.0016 (6)	−0.0002 (5)
C6	0.0194 (7)	0.0206 (7)	0.0206 (7)	−0.0015 (5)	−0.0038 (5)	0.0008 (6)
C7	0.0196 (7)	0.0193 (7)	0.0212 (7)	−0.0006 (5)	−0.0008 (6)	0.0012 (6)
C8	0.0178 (7)	0.0248 (8)	0.0207 (7)	0.0005 (6)	0.0000 (6)	0.0021 (6)
C9	0.0206 (7)	0.0189 (7)	0.0204 (7)	0.0017 (5)	−0.0001 (6)	0.0010 (6)
C10	0.0167 (7)	0.0170 (7)	0.0207 (7)	0.0023 (5)	0.0015 (5)	−0.0012 (6)
C11	0.0164 (7)	0.0222 (7)	0.0220 (7)	−0.0007 (5)	0.0032 (5)	0.0011 (6)
C12	0.0195 (7)	0.0215 (8)	0.0190 (7)	0.0001 (6)	0.0012 (5)	0.0008 (6)
C13	0.0161 (7)	0.0171 (7)	0.0228 (7)	0.0009 (5)	−0.0013 (5)	−0.0033 (6)
C14	0.0177 (7)	0.0187 (7)	0.0245 (7)	−0.0015 (5)	0.0039 (5)	0.0012 (6)
C15	0.0212 (7)	0.0186 (7)	0.0201 (7)	0.0016 (6)	0.0030 (5)	0.0015 (6)
C16	0.0213 (7)	0.0341 (9)	0.0235 (8)	0.0027 (6)	−0.0036 (6)	0.0014 (7)

### Geometric parameters (Å, °)

**Table:**

Cl1—C4	1.7431 (15)	C8—H8	0.9500
O1—C7	1.2240 (19)	C9—C10	1.464 (2)
O2—C13	1.3659 (18)	C9—H9	0.9500
O2—C16	1.4248 (19)	C10—C15	1.403 (2)
C1—C6	1.394 (2)	C10—C11	1.404 (2)
C1—C2	1.401 (2)	C11—C12	1.391 (2)
C1—C7	1.500 (2)	C11—H11	0.9500
C2—C3	1.384 (2)	C12—C13	1.395 (2)
C2—H2	0.9500	C12—H12	0.9500
C3—C4	1.392 (2)	C13—C14	1.395 (2)
C3—H3	0.9500	C14—C15	1.377 (2)
C4—C5	1.386 (2)	C14—H14	0.9500
C5—C6	1.393 (2)	C15—H15	0.9500
C5—H5	0.9500	C16—H16A	0.9800
C6—H6	0.9500	C16—H16B	0.9800
C7—C8	1.481 (2)	C16—H16C	0.9800
C8—C9	1.339 (2)		
			
C13—O2—C16	118.00 (12)	C8—C9—H9	116.2
C6—C1—C2	119.36 (14)	C10—C9—H9	116.2
C6—C1—C7	122.52 (13)	C15—C10—C11	118.00 (13)
C2—C1—C7	118.09 (13)	C15—C10—C9	118.70 (14)
C3—C2—C1	120.69 (14)	C11—C10—C9	123.25 (13)
C3—C2—H2	119.7	C12—C11—C10	121.06 (13)
C1—C2—H2	119.7	C12—C11—H11	119.5
C2—C3—C4	118.82 (13)	C10—C11—H11	119.5
C2—C3—H3	120.6	C11—C12—C13	119.52 (14)
C4—C3—H3	120.6	C11—C12—H12	120.2
C5—C4—C3	121.70 (14)	C13—C12—H12	120.2
C5—C4—Cl1	119.01 (12)	O2—C13—C12	125.02 (13)
C3—C4—Cl1	119.29 (11)	O2—C13—C14	114.83 (13)
C4—C5—C6	118.91 (14)	C12—C13—C14	120.15 (13)
C4—C5—H5	120.5	C15—C14—C13	119.82 (14)
C6—C5—H5	120.5	C15—C14—H14	120.1
C5—C6—C1	120.48 (13)	C13—C14—H14	120.1
C5—C6—H6	119.8	C14—C15—C10	121.41 (14)
C1—C6—H6	119.8	C14—C15—H15	119.3
O1—C7—C8	121.77 (14)	C10—C15—H15	119.3
O1—C7—C1	119.92 (13)	O2—C16—H16A	109.5
C8—C7—C1	118.31 (13)	O2—C16—H16B	109.5
C9—C8—C7	119.51 (14)	H16A—C16—H16B	109.5
C9—C8—H8	120.2	O2—C16—H16C	109.5
C7—C8—H8	120.2	H16A—C16—H16C	109.5
C8—C9—C10	127.64 (14)	H16B—C16—H16C	109.5
			
C6—C1—C2—C3	0.7 (2)	C7—C8—C9—C10	176.11 (14)
C7—C1—C2—C3	178.69 (13)	C8—C9—C10—C15	167.66 (15)
C1—C2—C3—C4	−1.0 (2)	C8—C9—C10—C11	−14.8 (2)
C2—C3—C4—C5	−0.1 (2)	C15—C10—C11—C12	1.4 (2)
C2—C3—C4—Cl1	179.45 (11)	C9—C10—C11—C12	−176.08 (14)
C3—C4—C5—C6	1.4 (2)	C10—C11—C12—C13	−1.4 (2)
Cl1—C4—C5—C6	−178.06 (11)	C16—O2—C13—C12	−7.9 (2)
C4—C5—C6—C1	−1.8 (2)	C16—O2—C13—C14	171.26 (13)
C2—C1—C6—C5	0.7 (2)	C11—C12—C13—O2	178.95 (14)
C7—C1—C6—C5	−177.17 (14)	C11—C12—C13—C14	−0.2 (2)
C6—C1—C7—O1	155.92 (15)	O2—C13—C14—C15	−177.58 (13)
C2—C1—C7—O1	−22.0 (2)	C12—C13—C14—C15	1.6 (2)
C6—C1—C7—C8	−24.0 (2)	C13—C14—C15—C10	−1.6 (2)
C2—C1—C7—C8	158.05 (14)	C11—C10—C15—C14	0.0 (2)
O1—C7—C8—C9	−17.5 (2)	C9—C10—C15—C14	177.68 (13)
C1—C7—C8—C9	162.41 (14)		

### Hydrogen-bond geometry (Å, °)

**Table:**

Cg1 and Cg2 are the centroids of the C1–C6 and C10–C15 rings, respectively.

**Table:**

*D*—H···*A*	*D*—H	H···*A*	*D*···*A*	*D*—H···*A*
C9—H9···O1	0.95	2.47	2.8080 (19)	101
C2—H2···Cg1^i^	0.95	2.85	3.4675 (15)	124
C12—H12···Cg2^ii^	0.95	2.92	3.6616 (17)	136

Symmetry codes: (i) *x*, −*y*+3/2, *z*+1/2; (ii) *x*, −*y*+3/2, *z*−1/2.
